# Is Conscious Stimulus Identification Dependent on Knowledge of the Perceptual Modality? Testing the “Source Misidentification Hypothesis”

**DOI:** 10.3389/fpsyg.2013.00116

**Published:** 2013-03-18

**Authors:** Morten Overgaard, Jonas Lindeløv, Stinna Svejstrup, Marianne Døssing, Tanja Hvid, Oliver Kauffmann, Kim Mouridsen

**Affiliations:** ^1^Cognitive Neuroscience Research Unit, Department of Communication and Psychology, Aalborg UniversityAalborg, Denmark; ^2^Cognitive Neuroscience Research Unit, Hammel Neurorehabilitation and Research Center, MindLab, Institute of Clinical Medicine, Aarhus UniversityAarhus C, Denmark; ^3^Research Centre Gnosis, Aarhus UniversityCopenhagen NV, Denmark; ^4^Laboratory for Somatosensory Testing and Research, University Hospital CopenhagenCopenhagen Ø, Denmark; ^5^Center of Functionally Integrative Neuroscience, MindLab, Aarhus UniversityAarhus C, Denmark

**Keywords:** consciousness, visual perception, auditory perception, multimodal integration, experience

## Abstract

This paper reports an experiment intended to test a particular hypothesis derived from blindsight research, which we name the “source misidentification hypothesis.” According to this hypothesis, a subject may be correct about a stimulus without being correct about how she had access to this knowledge (whether the stimulus was visual, auditory, or something else). We test this hypothesis in healthy subjects, asking them to report whether a masked stimulus was presented auditorily or visually, what the stimulus was, and how clearly they experienced the stimulus using the Perceptual Awareness Scale (PAS). We suggest that knowledge about perceptual modality may be a necessary precondition in order to issue correct reports of which stimulus was presented. Furthermore, we find that PAS ratings correlate with correctness, and that subjects are at chance level when reporting no conscious experience of the stimulus. To demonstrate that particular levels of reporting accuracy are obtained, we employ a statistical strategy, which operationally tests the hypothesis of non-equality, such that the usual rejection of the null-hypothesis admits the conclusion of equivalence.

## Introduction

The experiment, reported and discussed below, will test a possible dissociation between conscious access to the content of a visual or auditory perception, and access to information about perceptual modality. Intuition and some theories about conscious perception predict that a correct representation of content crucially involves knowledge about whether this content was visual or auditory. However, other theories, specifically relating to the study of blindsight, defend a “source misidentification hypothesis” according to which stimulus and modality identification must rely on independent processes. Resolving this question will have an important impact on several aspects of consciousness research, particularly those studying relations between conscious and unconscious cognition.

Recent years have seen a great increase in experimental investigations of how different cognitive and neural processes relate to conscious experience. However, as recognized in the field, there are certain methodological difficulties when trying to pin down such neural correlates (Metzinger, 2000; Hohwy, 2007). For one thing, it is very difficult to agree on a good candidate for a contrast in experiments using subtraction methods such as fMRI, ERP, and cognitive-behavioral measures. Based upon the assumption of pure insertion, the goal is to identify a *pure contrast* in which all cognitive, emotional, and behavioral processes are identical in the control and interest condition, except for those specifically related to conscious experience (Overgaard, 2011). Two well-studied examples are the phenomena of blindsight and subliminal perception in healthy individuals (Overgaard, 2012). Both cases have been suggested as possible “pure contrasts” as they are defined as performance above chance in the absence of conscious experience.

In recent discussions, the nature of both contrasts has been challenged. A number of experiments indicate that for both blindsight and subliminal perception, conscious experience, and correct reports about visual stimuli seem to correlate very well (Overgaard et al., 2008; Sandberg et al., 2010; Overgaard and Sandberg, 2012). In other words, there are empirical reasons not to think of the two cases as “contrasts” in the sense above.

In most cases, of course, subjective and objective reports go together (Del Cul et al., 2007), yet important differences in interpretation arise in those cases where they do not. In recent publications, we find more integrative interpretations that do not directly go against subliminal perception and blindsight as contrasts between conscious and unconscious processes, but do go against them as “pure.” For instance, Aru, Bachmann, Singer, and Melloni (Aru et al., 2012) show how a classical view of neural correlates of consciousness as the results of contrasts between conscious and unconscious conditions should be considered as much more complex, involving neural activations prior to and following the “moment in time” conscious experience occurs. Kouider and Dehaene (2007) also suggest that a clear distinction between “conscious” and “not conscious” might be too superficial and provide evidence to support “preconscious” processes. Both perspectives suggest in the present context that blindsight might not necessarily illustrate a “simple” contrast between conscious and unconscious perception, and that the exact nature of the preserved perceptual functions in blindsight is still unknown.

Most experiments that suggest subliminal perception rely either on dichotomous reports (“did you see it yes or no”?) or scales with arbitrary numbers of scale points (Sergent and Dehaene, 2004). One methodological innovation is the technique of discussing subjective ratings with the subject prior to the experiment, which yields greater variation in the clarity of reported conscious experiences, as in the Perceptual Awareness Scale (PAS) (Soto et al., 2011). The PAS is a four-point scale containing the following classifications: (CI) “clear image”, (ACI) “almost clear image” (WG) “weak glimpse”, and (NS) “not seen”. Using this method, we have shown that subliminal perception effects, obtained in the same experimental paradigm with a dichotomous report, fully disappear (Overgaard et al., 2006, 2008, 2010). The experiments indicate that at least some findings of unconscious visual identification abilities are the result of insensitive measures of conscious experience, and that more accurate measure of conscious experience show a more reliable correlation with objective task performance (Overgaard et al., 2006).

This methodologically rooted discussion has far reaching consequences as much of cognitive neuroscience theory is heavily influenced by the idea that most of our mental events, even complex events with semantic contents (Marcel, 1983), occur in the *total absence* of conscious experience and that self-reports in general are highly fallible. One defense of blindsight and subliminal perception taken as instances of visual abilities (e.g., stimulus identification) in the total absence of conscious experience is to say that although conscious experiences may be associated with visual stimuli, these experiences are not in themselves visual (Weiskrantz et al., 1995; Cowey, 2004; Kauffmann, 2009; Brogaard, 2011; Kauffmann, forthcoming). Such a view argues for an empirical separation between the neural representation of knowledge of the presented stimulus versus knowledge of which sensory modality has actually perceived the presented stimulus. The view is here named the “source misidentification hypothesis.” The hypothesis would in principle allow experiences of visual (or auditory) objects, which subjects however do not experience *as* visual (or auditory). Logically, the hypothesis rests on three assumptions: first that a decision regarding the modality responsible for perceiving a stimulus is cognitively accessed in a different way or by a different process than is the stimulus itself, second that the two processes are independent and dissociable so that we are able to know about the perceptual modality without the ability to identify a stimulus and third, most crucially that we are able to identify a stimulus without any knowledge about which modality actually perceived this stimulus.

Regarding the first assumption, although it may be true, it is not backed by any existing empirical evidence. Regarding the second, it consists of two dissociations. Whereas the first dissociation intuitively seems plausible, the second – that we might correctly identify a stimulus presented to us without knowledge of how it was perceived – is particularly counter-intuitive, as we are apparently almost always readily capable of issuing true reports about a sensory modality if questioned. Nevertheless, this is the crucial assumption upon which the defense is resting.

The assumption is however empirically testable. It is an empirical matter whether (1) the ability to identify the perceptual modality correlates with conscious experience just as stimulus identification does according to previous research (Overgaard et al., 2006), and (2) whether there are significant interactions between correct identifications of stimuli and of modality (Kauffmann, 2009).

Thus, two mutually exclusive hypotheses can be contrasted: from an “intuitive” standpoint, it would be expected that the correct identification of stimuli is dependent on the correct identification of perceptual modality (and perhaps even vice versa). Based on the previous studies using PAS, it may furthermore be expected that correct identifications correlate with reports regarding the clarity of conscious perception. The source misidentification hypothesis, however, would expect that the two identification tasks are independent *and* that they both may exist subliminally.

Below, we report an experiment contrasting these two hypotheses.

## Materials and Methods

The experiment was conducted according to principles in the Declaration of Helsinki and the local Ethical Committee for Northern Denmark. According to the local committee, no ethical application was necessary for this type of study.

Verbal consent was obtained from each participant, which by local ethics is considered sufficient for behavioral, non-biomedical studies. Work was carried out directly under the ethics committees’ requirements.

Fourteen students from Aalborg University participated voluntarily in the experiment (seven females, seven males; mean age, 23.3 years). One male subject was however excluded as he misunderstood instructions. All reported normal hearing and normal or corrected-to-normal vision with no history of neurological or psychiatric problems. All subjects signed an informed consent.

### Apparatus

The subjects were seated in a dimly illuminated room at a table facing a 13.3″ LED screen (resolution 1280 × 800). Visual stimuli were presented on a gray background on a monitor at 60 Hz placed 70 cm in front of the subjects’ eyes. Stimuli were presented using PsychoPy version 1.61 (Peirce, 2007). Auditory stimuli were adjusted on latency and loudness using Audacity.

### Stimuli and noise

Stimuli consisted of four vowels, “A,” “E,” “U,” and “Y.” These vowels were selected in pilot studies, where they demonstrated equal visual and auditory detection thresholds when presented in a mask of noise. Visual letters extended 1° and were presented for 333 ms centrally on the monitor. They were capital white letters of the Arial font type. Auditory letters had a duration of circa 100 ms and were presented from headphones. They were obtained from translate.google.com using a female voice. The experiment ran on a MacBook Pro 2.4 GHz Core2 A1278 and visual stimuli were presented on a 13.3″ 60 Hz monitor. Auditory letters had a duration of 100 ms and were presented from Sennheiser HD 25-1 11 headphones. They were synchronized using the software Audacity, and were presented as “the names of the letters” rather than the sounds. The auditory stimuli were mono, 44100 Hz, 32-bit float.

Prior to the experiment, all participants were introduced to the stimuli without masking. Hereafter, they could try the experiment and discuss matters of doubt with the experimenter before the actual experiment started.

All visual stimuli were superimposed on a mask. The visual mask consisted of random 1 pixel black and white dots, with a Gaussian transparency mask with three standard deviations at 3° radius. The mask was updated every frame. The auditory mask was brown noise with a power decrease of 6 dB per octave.

### Trial procedure

**Figure 1:**
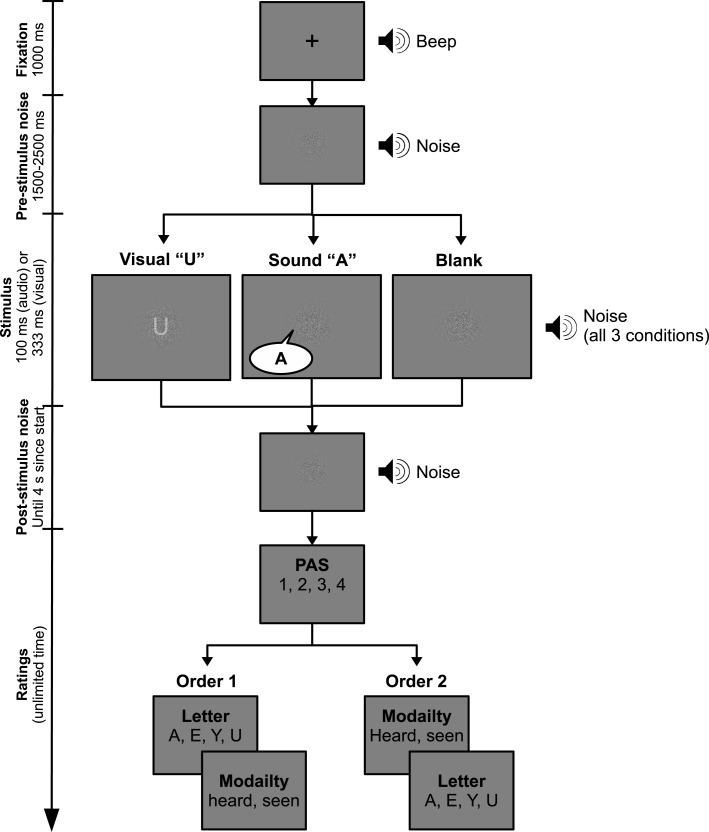
**Experimental trial procedure**. A fixation cross and a beep were presented 1s prior to every trial. Both masks were presented simultaneously for 3 s. Stimuli were superimposed on the mask at a random time between 1.5 and 2.5 s after mask initiation. Subjects reported experienced clarity using PAS, stimulus content, and perceptual modality after each trial.

The experimental procedure is illustrated in Figure 1. Only one stimulus (either visual or auditory) was presented in each trial. Subjects answered three questions regarding the stimulus. First, they rated the clarity of their experience of the stimulus using the PAS scale and keys “1”–“4” representing PAS-NS-CI. Second, they answered which stimulus they perceived, using buttons “A,” ”E,” “U,” and “Y.” Third, they answered in which modality the stimulus was presented, using button “8” for “heard it” (audio) and “0” for “saw it” (visual). The order of the latter two questions alternated from block to block, i.e., within subjects. The procedure is illustrated in Figure 1.

We decided to keep the order of the PAS rating and the identification task fixed as previous pilot experiments with PAS and highly similar paradigms showed no effect of this. Rather, an alternation might increase wrong responses, as subjects can be confused about which question they are answering when responding quickly.

The experiment included nine blocks of 20 trials each. There were 60 visual trials, 60 auditory trials, and 60 blank trials, which were presented in random order. Participants were not informed about the presence of blank trials. They served the function of a “control condition” to test whether subjects, as expected, reported “PAS = NS” in the absence of a stimulus.

### Instructions

Participants were instructed in the use of PAS. Subjects were encouraged to take their time to consider their response and use their intuition when reporting stimulus and modality. Subjects initially practiced on 24 auditory and visual trials with gradually declining stimulus intensities. Any confusion was discussed with the experimenter.

### Adjustment

The purpose of the adjustment was to find stimulus intensities that were slightly above each subject’s threshold, thus eliciting mostly PAS-NS and PAS-WG responses. Following the introduction, stimulus intensities were adjusted so that the average PAS responses for each subject were in the range of 1.25–1.75 (“1” being NS and “2” WG) within each modality. Visual intensity was adjusted using opacity and auditory intensity was adjusted using volume. Visual and auditory trials were presented in random order and the program evaluated the average PAS response every five visual trials and every five auditory trials. If the average was within the desired range, the modality-specific intensity was recorded. If the PAS-average was out of range, the modality-specific intensity was adjusted appropriately, weighted by the deviation from the desired average. The adjustment continued until at least two intensities were recorded for each modality. The program then proceeded to the actual experiment, using the average of the recorded intensities for each modality. Between 46 and 177 trials were required for a successful adjustment.

### Statistical analyses

One of our main hypotheses concerns a demonstration that the probability of correct report of stimulus and modality is at base-chance if NS is reported on PAS. The fundamental maxim in statistics is to establish evidence against a hypothesis, i.e., evidence is based on falsification of the hypothesis. Assuming a particular value for probability of correct report such as 25 or 50%, usual statistical practice is to attempt to reject this value with the observed data. In our case, however, we have the opposite interest, namely testing whether the data is consistent with a certain probability value. Effectively, we would like to accept the null-hypothesis. However, accepting a hypothesis based on observed data is problematic because the probability that our decision to accept the hypothesis is incorrect is unknown. In contrast, the probability of rejecting a true hypothesis, i.e., the probability of a false rejection, is fixed when the test is performed, and the probability level is decided by the experimenter, with 1 or 5% being the typical values. Hence it is known, that there is a 1 or 5% risk of falsely rejecting a hypothesis, which is the so-called type I error. On the other hand, accepting a hypothesis, which is indeed false, is an error of type II, and the probability of committing this incorrect decision is unknown for the particular test. Indeed, it is directly related to the test power (one minus the power), but *post hoc* power analysis is not meaningful.

We can apply standard statistical reasoning by reformulating the hypothesis. Instead of testing the hypothesis claiming that the probability of correct report is 50%, we test the hypothesis that this probability differs from 50% by a certain margin. If this new hypothesis can be rejected given the observed data, usual statistical practice permits the conclusion that the probability of correct report is chance level, to within the margins in the hypothesis. Since this procedure is now based on falsification, we reobtain the usual protection against a false decision at a prespecified level such as the 5% level. Although this is an “omnibus” approach to assess equivalence, the details of the implementation depends on the particular statistical test. Discussions of this approach in the setting of bioequivalence trials have been the focus of other recent publications (Berger and Hsu, 1996).

In this work we examine the relations between PAS and correctness of stimulus and modality reports using logistic regression. Therefore we need to implement the non-equivalence approach for this setting. Since multiple reports were obtained for each subject, we employ a mixed-model approach with a random effect for subject, to account for the correlation between reports from the same subject. We also included a time component to assess whether the effect of PAS was confounded by a learning effect.

A test for whether probability of correct stimulus report takes a certain value or lies in a certain interval corresponds to testing equivalent hypotheses for the regression (or beta-) coefficients in the logistic regression. These tests are based on the distribution of the coefficient under the null-hypothesis, which for the usual hypothesis beta = 0 is a *t*-distribution. The additional variation due to estimation of the random effect is accounted for by adjusting the degrees of freedom (Pinheiro and Bates, 2000). The test of non-equivalence can be formulated as the probability being outside the interval (50%−margin; 50%+ margin), i.e., the probability differs from 50% by more than a specified tolerance margin. A straightforward approach to testing the interval hypothesis is to test two one-sided hypotheses (i) probability < (50%−margin) and (ii) probability > (50%+ margin). If both (i) and (ii) can be rejected at the 5% level, then we may conclude that the probability is within the interval (50%−margin; 50% + margin). Since the non-equivalence hypothesis is the union of the one-sided tests (i) and (ii), it follows from the intersection-union (IU) principle (Berger and Hsu, 1996) that the size of the overall test is equal to the size of the individual tests. Therefore, we test the non-equivalence hypothesis by testing the two one-sided hypothesis (i) and (ii) at level alpha = 5%. We take the *p*-value to be the largest *p*-value for the one-sided tests. We illustrate the application of this test using a margin of 10% centered on chance level, although we note that that the interval does not need to be symmetric.

Since most statistical software packages do not readily provide the one-sided tests with non-zero values, we note that the non-equivalence can be performed using the two limits of the 90% confidence interval (Berger and Hsu, 1996). If the lower limit of the 90% CI is below the downward limit of the equivalence region, then the alpha = 5% level *t*-test for (i) cannot be rejected. Analogously, if the upper limit of the 90% CI exceeds the upward boundary of the equivalence region, then (ii) cannot be rejected. Since the reverse implications are also true, we can reject the interval hypothesis and declare equivalence if and only if the 90% CI is fully contained in the equivalence region. Thus there is an operational equivalence (Berger and Hsu, 1996) between the test of the two one-sided tests (i, ii) and the 90% CI procedure. However the 90% CI should be considered only as a practical test “instrument” leading to a dichotomous decision (whereas the actual tests in (i) and (ii) yield a *p*-value). There is no conceptual overlap with the interpretation of the CI as covering the true parameter value with a certain probability. Confidence intervals are calculated throughout the manuscript as parameter estimate ± the standard error multiplied by the relevant quantile of the null-distribution.

To assess the stochastic dependence between correctness of modality and stimulus report, we used a loglinear model and a bivariate logistic regression with PAS level as a modulator for both outcomes as well as their odds ratio. The latter model admits a formal test for equal dependence between stimulus and modality report at different levels of PAS. Analyses were performed in R version (2.10.1), with packages MASS version 7.3-17 and lme4 version 0.999375-42.

Inference in the logistic regression and loglinear models is based on *t*-tests and likelihood ratio tests (chi-squared tests). The Wilcoxon signed rank test is applied to test for difference in sensitivity and specificity in modality identification at PAS = NS and PAS = WG. A bivariate logistic regression model is used to assess the dependence between performance in modality and stimulus identification at different PAS levels.

## Results

**Figure 2:**
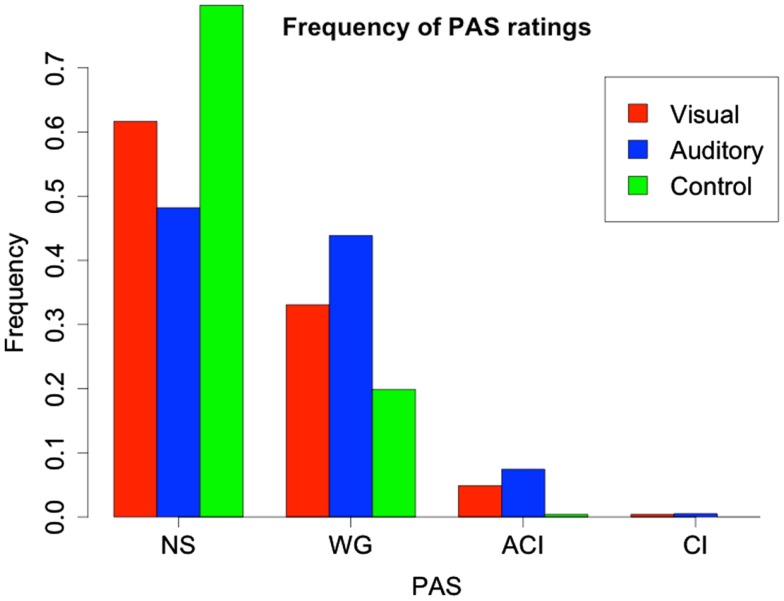
**Frequency of PAS reports for visual, auditory, and control trials**. We could only analyze NS and WG type responses, as ACI and CI were very infrequent due to the adjustment procedure. As expected, the great majority of control trials were rated NS.

The average stimulus intensities obtained in the calibration was 97.6 ± 0.5% transparency for visual stimuli and 17 ± 4% volume for auditory stimuli across subjects. As the adjustment procedure aimed for PAS reports to be in the range of 1.25–1.75 (“1” being NS and “2” WG), an illustration of the frequency of the PAS categories show that NS and WG have been used almost exclusively (see Figure 2).

Subjects reported which letter was presented (one of four letters), experienced perceptual clarity using PAS, and perceptual modality (auditory or visual). We employed a mixed-model logistic regression with ”correct/incorrect” as outcome and a random effect for subject. In this way, as in previous papers, we used PAS as predictor of another variable to study their relation.

**Figure 3:**
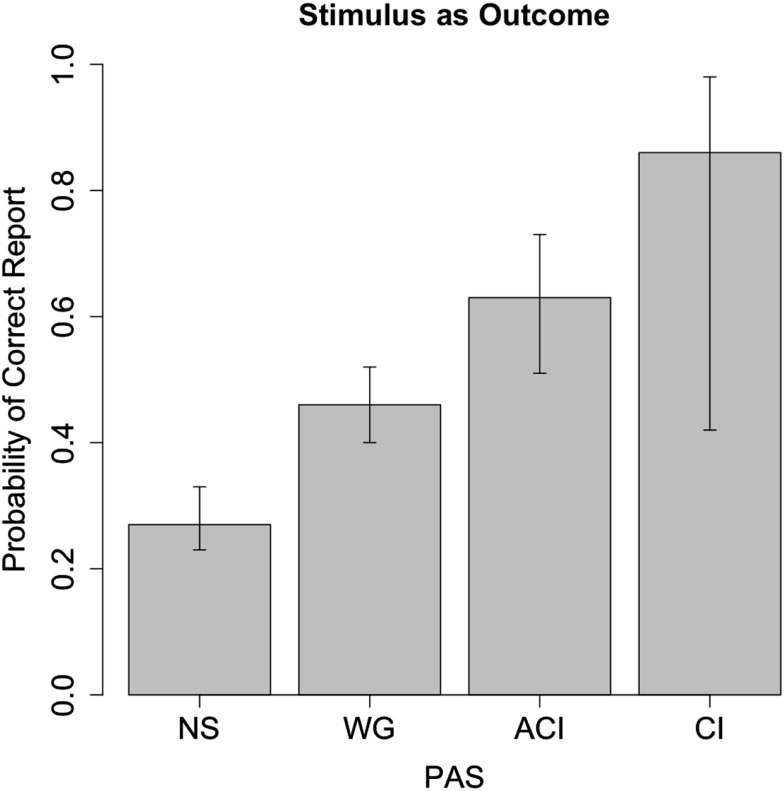
**Correctness of stimulus report as a function of PAS**. The likelihood of correct stimulus report when PAS = NS (27%) is close to the chance level of 25%, whereas higher degrees of perception correlate with progressively higher accuracy.

The learning effect was not significant [*t*(1543) = 1.43, *p* = 0.15] and was not included in further analyses. In the logistic regression model fitted across all trials with correctness of stimulus report as outcome and PAS as predictor and a random effect for subject, the probability of correct report when PAS = NS was 0.27, with ± one standard error interval (0.25, 0.30) and 95% confidence intervals (0.23; 0.33), suggesting there is no indication of subliminal perception since the estimate is approximately chance level (0.25) with little variation around this value (see Figure 3). As a formal assessment, we tested the hypothesis that the probability of correct report is <0.15 or >0.35 using the IU principle as described above. As *p* = 0.003 [*t*(1544) = −2.79], we can reject the hypothesis that the probability of correct report is outside the interval (0.15; 0.35). Equivalently, we see that the 90% CI is (0.23; 0.32) is fully contained in the equivalence region, leading to a rejection of the null-hypothesis of non-equivalence. The narrowness of the CI suggests that performance could be determined to be equivalent to chance level within a smaller margin than 10%.

The probability of correct stimulus identification as PAS = WG is 0.46, SE interval (0.43, 0.49), which is significantly different from PAS = NS, *p* < 0.001 [*t*(1544) = 7.07]. This corresponds to an odds ratio of 2.25 with confidence interval (1.80; 2.82) indicating a substantial increase in correctness when PAS = WG compared to NS. The standard deviation of the random effect for subject was 0.36 with 95% CI (0.22, 0.60). This suggests some response bias across subjects, with some subjects consistently being more correct than others. The 95% CI corresponds to a subject variation between 21 and 35% in stimulus correctness at PAS = NS.

In a similar model, with correctness of modality report as outcome and PAS as predictor, it is shown that the probability of correct modality report when PAS = NS is 0.52, SE interval (0.49, 0.55), with 95% confidence interval (0.47; 0.58). This is, again, very close to chance level (0.5). The hypothesis prob < 0.40 or prob > 0.60 is rejected with *p* = 0.004 [*t*(1544) = −2.68] suggesting the probability of correct modality identification is equivalent to 50% within a 10% margin. The 90% CI is [0.47; 0.57] which indicates the actual equivalence region may be smaller. The learning effect was not significant [*t*(1543) = 0.12, *p* = 0.90] and was omitted from the analyses.

There is a significant difference between identification correctness for PAS = NS and PAS = WG, *p* < 0.001 [*t*(1544) = 10.86], corresponding to an odds ratio of 3.84, CI (3.01; 4.90).

The standard deviation of modality correctness at subject level (response bias) for PAS = NS was 0.34 with 95% CI (0.20, 0.58), corresponding to an interval of between 44 and 61%.

**Figure 4:**
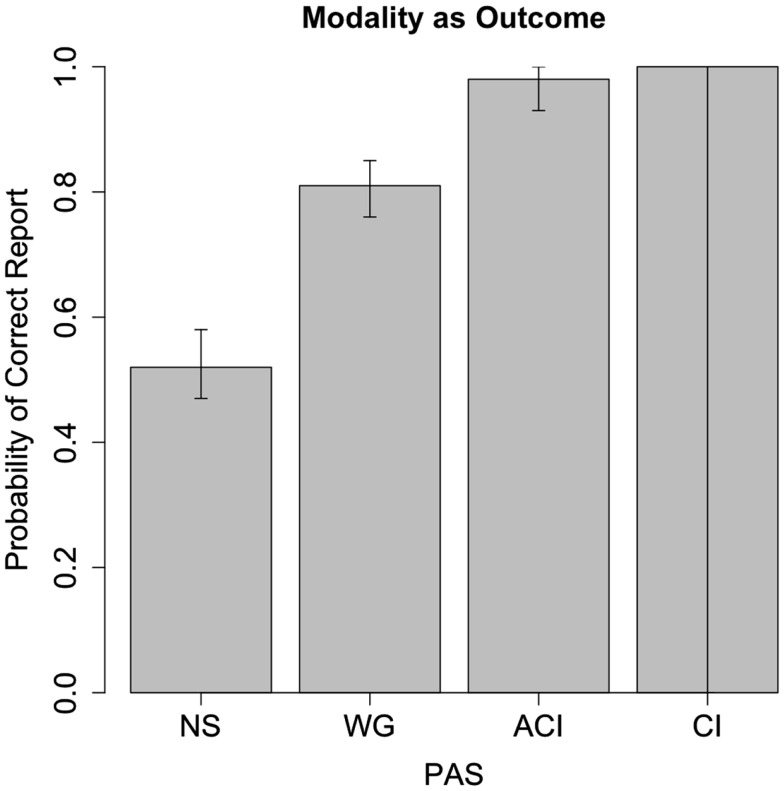
**Correctness and 95% CI for modality report as a function of PAS report**. Correctness of modality report is associated with degree of perception, with correctness at 52% for PAS = NS, which is close to chance level (50%). The broadness of the 95% CI at PAS = CI ensues from the sparsity of observations due to the calibration procedure (see Figure 2).

As shown in Figures 3 and 4, PAS predicts performance in both models, so that, at PAS = NS, subjects have neither subliminal access to information about the stimulus or the correct modality. It seems conscious experience, however vague, is a necessary precondition for the ability to correctly identify stimulus as well as modality. We note that the confidence interval for modality correctness is very broad for PAS = CL, which we believe results from very few (seven) reports, which produces statistical uncertainty about the parameter estimate.

**Figure 5:**
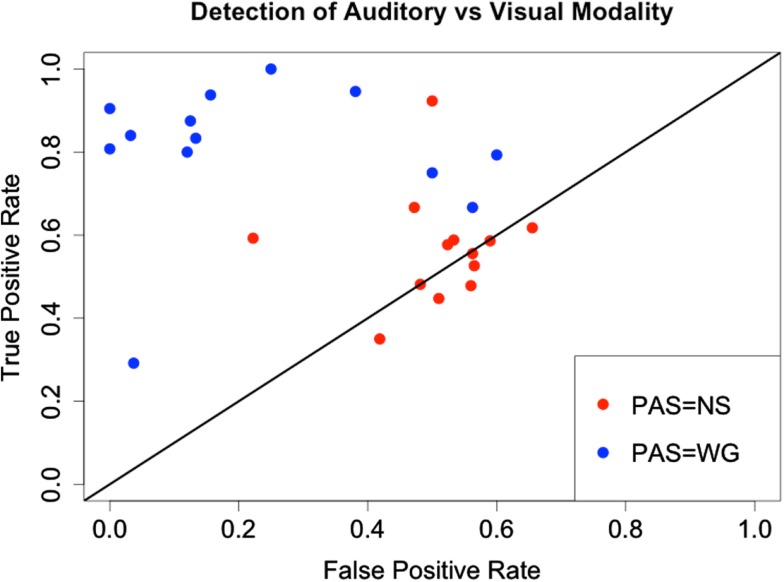
**True positive and false positive rates for the detection of auditory stimulus versus visual stimulus for the *N* = 13 subjects**. At PAS = NS points center on the identity line and with true and false positive rates around 0.5, supporting the hypothesis that no knowledge of modality is available. At PAS = WG comparatively more information seems to be accessible, as suggested by the higher true positive rate and lower false positive rate.

To further assess a PAS-dependent ability to identify auditory relative to visual stimulus, we calculated sensitivity and specificity for the modality reports. We use as sensitivity the probability of correctly reporting modality as auditory in auditory cases, and specificity the probability of correctly reporting modality as visual in the visual cases. In Figure 5 we plot the sensitivity (true positive rate) against the false positive rate (one-specificity) to parallel a receiver operator characteristics (ROC) curve, where points close to the identity line indicate no predictive ability in contrast to points in the upper left corner with high discriminatory performance. Indeed for PAS = NS points cluster on the middle of the identity line corresponding to 50% probability of correct identification in both auditory and visual cases. At PAS = WG there is a clear trend toward high true positive rate and low false positive rate, indicating substantially improved discriminatory power, however with four subjects demonstrating a tendency to underperform on the visual task (false positive rate exceeding 30%). The increase in sensitivity as well as decrease in false positive rate when PAS = WG instead of NS, are both significant [one-sample Wilcoxon test, *V* = 8, *p* = 0.006, (sensitivity), *V* = 88, *p* = 0.001 (false positive rate)].

A loglinear model with response frequency as a function of PAS level (NS and WG) and correctness of modality and stimulus response shows a significant dependence between the three factors (χ^2^ = 15.75, *df* = 1, *p* < 0.001). However, the dependence between correctness of stimulus and modality report is modulated by PAS. At PAS = NS correctness of modality and stimulus are independent (χ^2^ = 2.20, *df* = 1, *p* = 0.14) whereas at PAS = WG they are dependent (χ^2^ = 35.03, *df* = 1, *p* < 0.001). In other words: (a) Without conscious experience, modality and stimulus correctness do not modulate each other and (b) they do modulate each other when there is weak conscious experience.

A bivariate logistic regression with stimulus and modality correctness as outcome and PAS as predictor shows a significant difference in odds ratios between stimulus and modality correctness at PAS = NS, OR = 1.25, and PAS = WG, OR = 3.75, *z* = −3.92, *p* < 0.001. This change in odds ratio by a factor of three suggests a substantially stronger dependence between correctness of stimulus and modality report at PAS = WG compared to PAS = NS.

**Figure 6:**
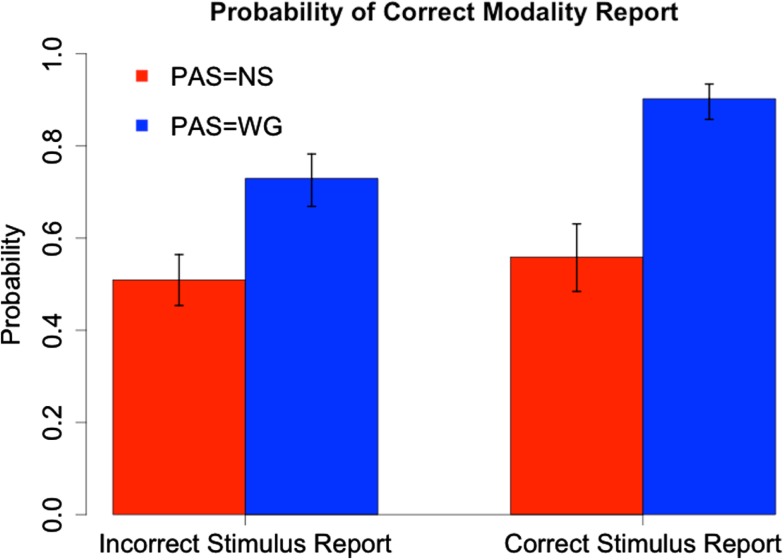
**Probability of correct modality report as a function of PAS and correctness of stimulus report**. Ninety-five percent confidence intervals for estimated probabilities are based on combined standard errors of the estimates of fixed effects and interactions.

**Figure 7:**
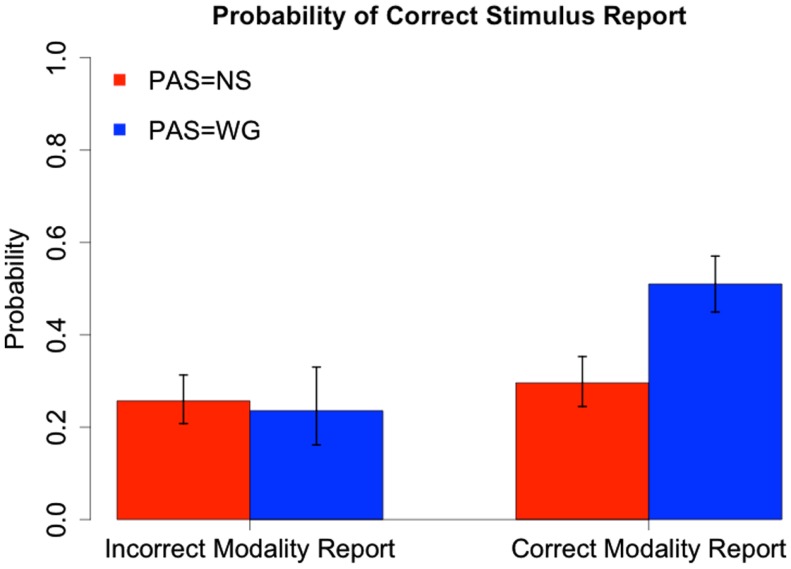
**Probability of correct stimulus report given correctness of modality report and PAS**. Ninety-five percent confidence intervals for estimated probabilities are based on combined standard errors of the estimates of fixed effects and interactions.

We illustrate the combined effects of PAS with stimulus and modality identification in Figures 6 and 7. Based on a logistic regression with PAS and stimulus identification as predictors of modality (Figure 6) and PAS and modality identification as predictors of stimulus (Figure 7), we see that at PAS = NS there is little difference in probability of correct report of modality (stimulus) when stimulus (modality) is identified correctly instead of incorrectly. In contrast, at PAS = WG there is a 19% points increase in probability of correct modality identification and 29 percentage points increase in correct stimulus identification when stimulus (modality) is correctly identified. There are significant interactions between PAS and correctness of modality report (χ^2^ = 13.77, *df* = 1, *p* < 0.001) and between PAS and correctness of stimulus report (χ^2^ = 13.32, *df* = 1, *p* < 0.001).

The two analyses with correctness of modality and stimulus as, respectively, predictor, and response, suggest the difference in probability at PAS = NS and WG of correct modality identification is different from the increase in probability of correct stimulus identification, although this could not be directly tested. This necessitates having correctness of modality and correctness of stimulus as response in the logistic regression, but with the same variables as predictors (correctness of stimulus predicting correctness of modality and vice versa). This would lead to the same data point being used twice, one time with modality as response and stimulus as predictor, and one time where these roles are switched. To avoid this, we split the trials into two halves, considering modality as predictor for stimulus in the one half and opposite in the second half. The logistic regression model now has three independent variables: “question” type (modality/stimulus), correctness of reply to this question and PAS level.

We fit a logistic regression model with subject as a random effect and modulating question (modality/stimulus), correctness of reply to this question, and PAS level as fixed effects, predicting the correctness of the “other” question (stimulus/modality). We find no three-way interaction between question, its correctness and PAS (χ^2^ = 0.40, *df* = 1, *p* = 0.53). Neither is there an interaction between question and its correctness (χ^2^ = 0.46, *df* = 1, *p* = 0.67). However, we do see an interaction between PAS and question (χ^2^ = 13.53, *df* = 1, *p* = 0.0002), and between PAS and correctness of reply to the question (χ^2^ = 13.29, *df* = 1, *p* = 0.0003).). This suggests the difference between PAS = NS and WG for modality and stimulus reporting is different. Clearly the probability of correct report increases between the stimulus predictor and modality predictor since chance level is higher for modality report, however this analysis suggests the difference in report correctness between PAS = NS and WG for modality and stimulus report as predictors is further modulated by the interaction between PAS and predictor question correctness

In a subanalysis considering only cases where the predictor report was incorrect (the left bar groups in Figures 6 and 7) there was a significant interaction between PAS and report (χ^2^ = 9.11, *df* = 1, *p* = 0.003) whereas the PAS main effect was not (*z* = −0.49, *p* = 9.63), suggesting that PAS modulates modality correctness when stimulus is incorrect, but PAS does not modulate stimulus correctness when modality is incorrect.

## Discussion

Our experiment confirms that the correct identification of a stimulus co-varies with PAS rating. When subjects report not to see anything, they are at chance level, whereas they are significantly above chance whenever they report a “brief (visual) glimpse.” More surprisingly, this relationship is essentially the same for identification of modality. At least under the conditions of this experiment, knowledge of perceptual modality seems only to exist, or to be accessible, when there is at least a weak conscious experience. For modality reports, results may indicate that less information is necessary compared to reports about stimuli in order for subjects to be at or close to maximal correctness (see Figure 4 compared to Figure 3).

As a further note on methodology, we point out that to demonstrate equivalence between actual probabilities and fixed values we operationally “switched” the common null-hypothesis (true parameter equal to fixed value) and the alternative (true parameter different from fixed value). This ensures the risk of falsely declaring equivalence is bounded by the fixed significance level, alpha = 5%, and constitutes the FDA-recommended methodological procedure in equivalence trials (Berger and Hsu, 1996; US Department of Health, and Human Services, Food, and Drug Administration, 1999). Hypothesis tests are based on the same distributional characteristics of the test statistic as in the traditional null-hypothesis test, but with other critical regions. Importantly, the traditional confidence interval for the parameter of interest serves the dual purpose of quantifying confidence in the estimated parameter as well as being instrumental in testing equivalence. We stress that the use of the 90% CI in equivalence testing is purely operational, and may not necessarily apply to other types of equivalence (Berger and Hsu, 1996) tests.

In the context of this experiment, and with the aid of this statistical approach, we demonstrate that the ability to identify modality above chance level seems dependent on PAS level. However, correct modality identification seems a necessary precondition for correct stimulus identification above chance level, regardless of PAS level. Thus, stimulus and modality identification seem not to be mutually independent processes.

This result goes against the source misidentification hypothesis, while it supports the intuition that reports about conscious experiences related to, e.g., a visual stimulus, are in fact also experienced and reported as visual when experience is sampled in an appropriately sensitive manner. As it would hardly be argued that such vague experiences are auditory or belong to some other sense, a competing position might argue in favor of some “modality free” representation of a stimulus with a non-sensory experience attached to it. Such an idea is however also rejected by these results, as stimulus identification does not occur above chance level scores in the absence of modality identification.

It is important to mention that we of course do not consider all possible conscious experiences sensory, and that the hypothesis here is tested in a rather narrow framework, comparing visual, and auditory perception only. Accordingly, it cannot be guaranteed that the results would generalize to other modalities or different stimulus material. However, the experiment does set up one case for which our competing hypotheses have strong and different expectations of the outcome. For this reason, one appears strengthened and the other weakened.

It is, also, not obviously the case that these results generalize to blindsight, although the hypotheses were derived from this area of research. Nevertheless, the interpretation of blindsight as a pure contrast in consciousness research crucially relies on the correctness of the source misidentification hypothesis. For this reason, further research with this particular focus could be of much value.

One might argue there is still one conceptual issue to discuss: which PAS scale points represent a lack of conscious experience? In the conceptual framework behind the experiment, as suggested in the introduction, consciousness is associated with any subjectively notable visual experience related to the stimulus. In consequence, for vision to be unconscious or subliminal, nothing related to the stimulus must be experienced. This is the case for PAS = NS only. A different interpretation of consciousness could be that the content of the stimulus must be identified for something to be conscious. In such a case, a visual experience of stimulation, in which the subject is unable to report the content of the stimulus, would not count as conscious perception. In such a case PAS = WG would also be unconscious, although this is not what is typically meant by the term (Metzinger, 2000; Cowey, 2004; Overgaard and Sandberg, 2012).

## Conflict of Interest Statement

The authors declare that the research was conducted in the absence of any commercial or financial relationships that could be construed as a potential conflict of interest.
